# Supplementary material for: Exploring the Phytobeneficial and Biocontrol Capacities of Endophytic Bacteria Isolated from Hybrid Vanilla Pods
Source: Microorganisms. 2023 Jul 5;11(7):1754. doi: 10.3390/microorganisms11071754 (PMC10385615; doi:10.3390/microorganisms11071754)
Supplement: Supplementary file 1 [file microorganisms-11-01754-s001.zip › microorganisms-2419312-supplementary.pdf]

Article

Exploring the phytobeneficial and biocontrol capacities of endophytic bacteria isolated from hybrid vanilla pods

Guillaume Lalanne-Tisné [1,*], Bastien Barral [1], Ahmed Taibi [1], Zana Kpatolo Coulibaly [1], Pierre Burguet [2], Felah Rasoarahona [3], Loic Quinton [2], Jean-Christophe Meile [1], Hasna Boubakri [4] and Hippolyte Kodja [1,*]

1 Qualisud, Univ Montpellier, Avignon Université, CIRAD, Institut Agro, IRD, Université de La Réunion, 15 avenue René Cassin 97744 ST-DENIS cedex 9
2 Mass Spectrometry Laboratory-MolSys, GIGA proteomics Facility, University of Liège, Liège, Belgium
3 Université d'Antananarivo, École Supérieure des Sciences Agronomiques, Département IAA, Madagascar
4 Université de Lyon, F-69361, Université Claude Bernard Lyon 1, CNRS, UMR 5557, INRAE UMR1418, Ecologie Microbienne, F-69622, Villeurbanne, France
* Correspondence: guillaume.lalanne-tisne@univ-reunion.fr; Hippolyte.kodja@univ-reunion.fr

Supplementary Figures

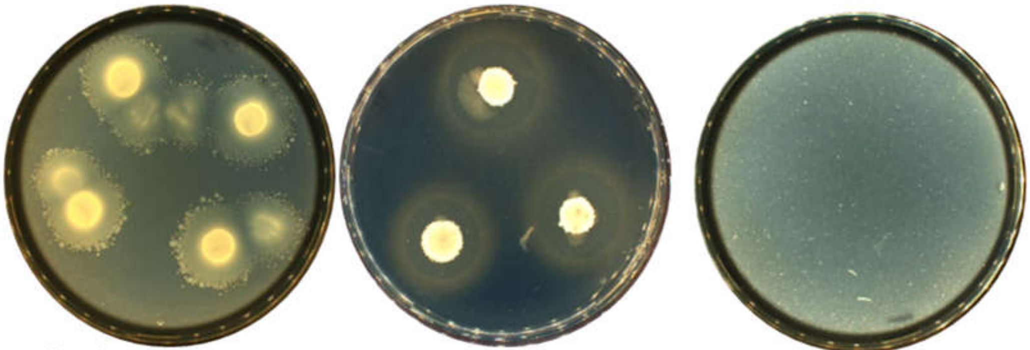

Figure S1. Halos produced by bacteria on MM9 – CAS agar by spot inoculation and incubation for 7 days at 28° C. (a) Pseudomonas fluorescens (F113) as positive control. (b) Bacillus subtilis (T2). (c) water as negative control. The halos around the colonies of bacteria indicating the ability of this isolate to extract siderophore that removes Fe from Fe-CAS agar medium.

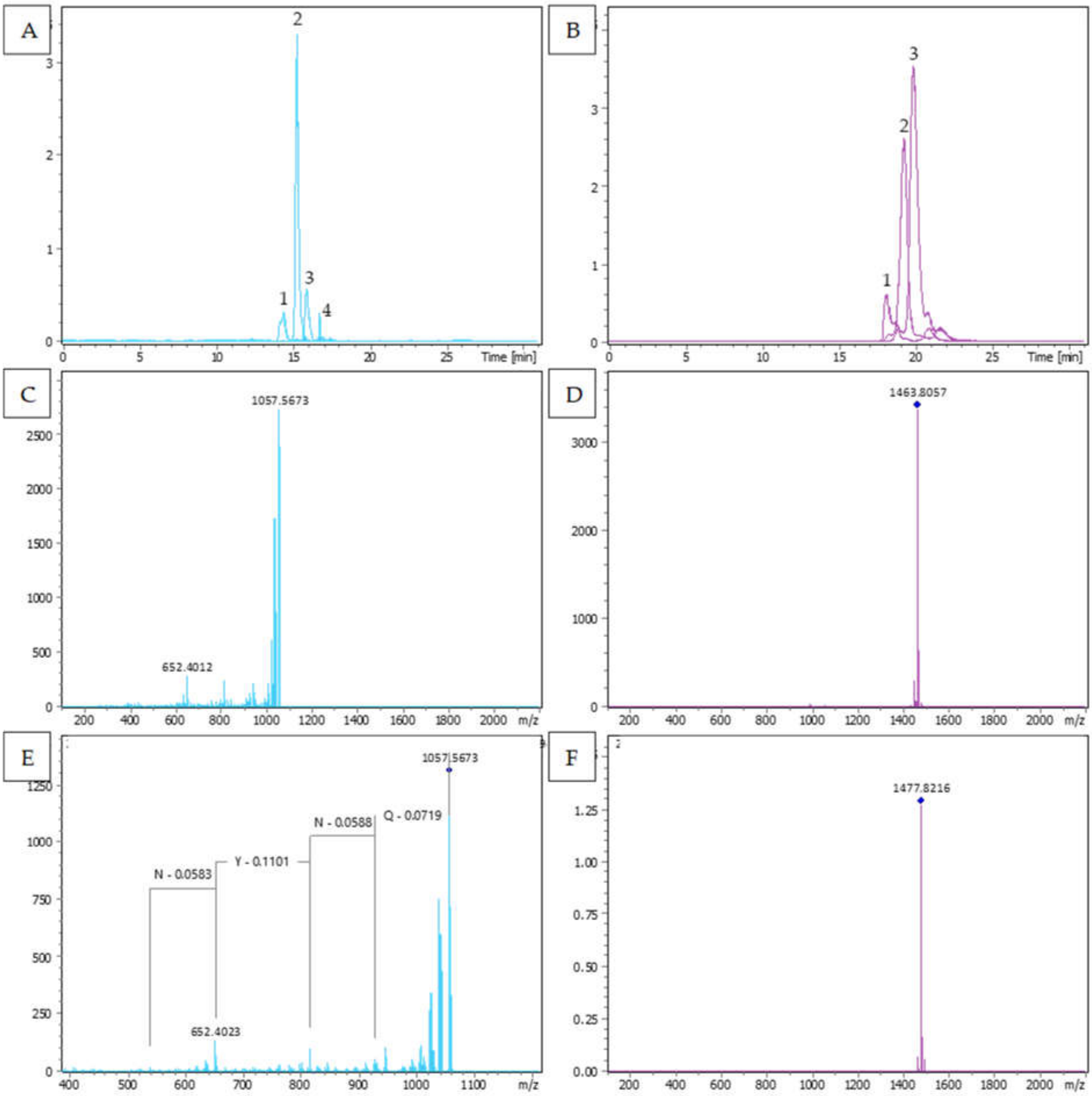

Figure S2. TIMS-TOF analysis of iturins and fengycins recorded in the inhibition area between *Bacillus siamensis* (m65) and *Fusarium oxysporum sp. Vanillae* (Fo72a). (A) The extracted ion chromatogram of 1043.5525 (1), 1057.56873 (2), 1071.5838 (3) and 1085.5995 (4) m/z representing the four present metabolites of iturins. (C) MS spectrum represented the single charged M+H+ ion of iturin 1057.56 m/z. (E) MSMS spectrum ion at 1057.56 m/z and the fragmentation lead to the peptide sequence analysis of iturin. (B) The extracted ion chromatogram of 1449.7891 (1), 1463.8057 (2) and 1477.8216 (3) m/z representing the three present metabolites of fengycin. (D) MS spectrum represented the single charged M+H+ ion of fengycin 1463.8057 m/z. (F) MS spectrum represented the single charged M+H+ ion of fengycin 1477.8216 m/z.

Data S1. Statistical data summary

1. **PGP capacities**
   a. <u>Phosphate Solubilizing Efficiency</u>

Kruskal-Wallis rank sum test

Kruskal-Wallis chi-squared = 76.319, df = 12, p-value = 2.068e-11

LSD test

```
$statistics
     Chisq Df      p.chisq  t.value      MSD
  76.31869 12 2.068123e-11 3.490679 47.75547

$parameters
            test  p.ajusted     name.t ntr alpha
  Kruskal-Wallis bonferroni pgp$strain  13  0.05

$means
     pgp.phosphate       rank        std  r          Min        Max
F113  0.4768573198 126.666667 0.09455701 12  0.358849518 0.62232373
m61   0.0004464558   9.541667 0.03079993 12 -0.045971546 0.05280921
m62a  0.4957948272 115.666667 0.26582415 12  0.069069954 0.92654445
m62b  0.2545417353  71.083333 0.19702949 12  0.019818694 0.70551871
m64   0.4025251804 100.500000 0.22956854 12  0.129357623 0.85348500
m65   0.4339810917 101.583333 0.28037709 12  0.163873410 0.98036221
m67   0.2303907319  68.291667 0.14469250 12 -0.028187911 0.42047359
m72   0.0546913977  24.166667 0.04248442 12 -0.004840512 0.16054687
t17a  0.2498678024  72.833333 0.11884071 12  0.053901410 0.43570470
t2    0.3153487185  88.083333 0.16567042 12  0.047286679 0.53972109
t24   0.3055572941  84.250000 0.17434036 12  0.103842447 0.67685524
t30   0.2240738036  68.000000 0.16804600 12  0.035436130 0.51317350
t5    0.3183188205  89.833333 0.05957723 12  0.227996167 0.42938934
             Q25         Q50        Q75
F113  0.39712368 0.448892441 0.57421554
m61  -0.02623273 0.001014618 0.01637896
m62a  0.35838925 0.425102951 0.66935872
m62b  0.13231456 0.223683196 0.34972195
m64   0.28502365 0.355194462 0.46318677
m65   0.19179291 0.353185464 0.61836887
m67   0.13332667 0.263247908 0.31953434
m72   0.03002767 0.044673758 0.07006715
```

```
t17a  0.18551508 0.287005042 0.32279965
t2    0.19089894 0.309974524 0.48151191
t24   0.15274556 0.276182365 0.42554173
t30   0.08495130 0.182489965 0.34546500
t5    0.25872135 0.333488567 0.35522864

$comparison
NULL

$groups
     pgp$phosphate groups
F113    126.666667      a
m62a    115.666667     ab
m65     101.583333     ab
m64     100.500000     ab
t5       89.833333     ab
t2       88.083333     ab
t24      84.250000     ab
t17a     72.833333      b
m62b     71.083333     bc
m67      68.291667     bc
t30      68.000000     bc
m72      24.166667     cd
m61       9.541667      d
```

b. Siderophore production

Kruskal-Wallis rank sum test

Kruskal-Wallis chi-squared = 93, df = 12, p-value = 1.293e-14

LSD test

```
$statistics
     Chisq Df      p.chisq
  93.00021 12 1.287859e-14

$parameters
            test  p.ajusted     name.t ntr alpha
  Kruskal-Wallis bonferroni pgp$strain  13  0.05

$means
     pgp.siderophore      rank       std  r       Min       Max       Q25
F113       1.1975036 101.62500 0.3667403  8 0.6239316 1.7482517 0.9692699
m61        0.0000000   8.00000 0.0000000 12 0.0000000 0.0000000 0.0000000
m62a       1.2903037 109.83333 0.3736990 12 0.8367580 1.9112051 0.9864072
```

```
m62b       1.2400503 104.50000 0.3747666 12 0.7864173 1.8587963 0.9562445
m64        1.0122403  82.50000 0.3185396 12 0.5650273 1.5061728 0.7315398
m65        1.3680499 108.41667 0.5148358 12 0.6300000 2.1584582 0.9548542
m67        0.9292265  76.50000 0.2342729 12 0.5176367 1.3977433 0.8131613
m72        0.4655038  28.50000 0.3040176 12 0.0000000 0.7577197 0.2275510
t17a       0.7294683  47.25000 0.1318999 12 0.5801847 1.0034423 0.6449307
t2         1.4014038 118.25000 0.3836919 12 0.9695757 2.1777137 1.0607176
t24        1.1654596 103.09091 0.2285207 11 0.8615385 1.6317992 0.9947637
t30        0.6891822  43.16667 0.1990041 12 0.3760933 1.0266160 0.5529083
t5         0.8734111  67.16667 0.1879161 12 0.7092050 1.3240059 0.7489852
           Q50       Q75
F113 1.2042917 1.4402928
m61  0.0000000 0.0000000
m62a 1.2055953 1.5604127
m62b 1.0821353 1.5918854
m64  1.0212465 1.2966741
m65  1.3694702 1.6635442
m67  0.9616970 1.0590354
m72  0.5937886 0.6948545
t17a 0.6955990 0.7732389
t2   1.2672123 1.6817934
t24  1.1080306 1.2982827
t30  0.6580087 0.8522706
t5   0.8192186 0.8884778

$comparison
NULL

$groups
     pgp$siderophore groups
t2         118.25000      a
m62a       109.83333     ab
m65        108.41667     ab
m62b       104.50000    abc
t24        103.09091    abc
F113       101.62500    abc
m64         82.50000   abcd
m67         76.50000    bcd
t5          67.16667    cde
t17a        47.25000    def
t30         43.16667    def
m72         28.50000     ef
m61          8.00000      f
```

c. <u>IAA production</u>

Kruskal-Wallis rank sum test

Kruskal-Wallis chi-squared = 44.426, df = 11, p-value = 6.12e-06

LSD test

```
$statistics
    Chisq Df      p.chisq
  44.4254 11 6.121242e-06

$parameters
            test  p.ajusted     name.t ntr alpha
  Kruskal-Wallis bonferroni pgp$strain  12  0.05

$means
        pgp.IAA      rank        std  r    Min   Max     Q25    Q50     Q75
m61  0.23484615  76.84615 0.23687826 13 0.0660 0.922 0.07800 0.1200 0.29100
m62a 0.14636364  76.22727 0.05681597 11 0.0730 0.216 0.10900 0.1220 0.20600
m62b 0.08542857  40.35714 0.04670647 14 0.0210 0.180 0.06350 0.0845 0.10425
m64  0.18585714  67.64286 0.18078413 14 0.0460 0.680 0.07450 0.1460 0.19575
m65  0.13485714  71.25000 0.04462469 14 0.0640 0.200 0.09625 0.1370 0.17675
m67  0.38810000  87.45000 0.48721897 10 0.0520 1.315 0.11675 0.1445 0.34250
m72  1.29941667 129.54167 0.70784866 12 0.0770 2.700 1.10200 1.3765 1.52075
t17a 0.49981818 113.36364 0.41330009 11 0.1000 1.262 0.23700 0.3120 0.62650
t2   0.15678571  52.78571 0.16691001 14 0.0370 0.583 0.07075 0.0845 0.11775
t24  0.12958571  60.92857 0.07434992 14 0.0432 0.282 0.07975 0.0935 0.19225
t30  0.42300000  85.16667 0.46404761 12 0.0440 1.304 0.10450 0.1380 0.58700
t5   0.11918182  62.54545 0.04104100 11 0.0630 0.189 0.08650 0.1240 0.14350

$comparison
NULL

$groups
       pgp$IAA groups
m72  129.54167      a
t17a 113.36364     ab
m67   87.45000    abc
t30   85.16667    abc
m61   76.84615     bc
m62a  76.22727     bc
m65   71.25000     bc
m64   67.64286     bc
t5    62.54545     bc
t24   60.92857     bc
```

```
t2    52.78571      c
m62b  40.35714      c
```

2. **Antagonism assay**
    a. Against *C. orchidophilum*

Kruskal-Wallis rank sum test

Kruskal-Wallis chi-squared = 99.636, df = 13, p-value = 1.952e-15

LSD test

```
$statistics
     Chisq Df      p.chisq
  99.63592 13 1.998401e-15

$parameters
            test  p.ajusted    name.t ntr alpha
  Kruskal-Wallis bonferroni df_co$ind  14  0.05

$means
       df_co.inhib      rank       std r         Min         Max        Q25
C-    1.096032e-14  30.88889  2.322976 9  -2.7418455  4.1893096 -2.0055301
C+    4.179163e+01 112.83333  9.357089 6  33.9398914 54.1864576 35.4855880
m61   5.091797e+00  44.00000  5.622360 9  -1.5599517 15.6153805  0.3738908
m62a  1.649792e+01  69.12500  8.988384 8   6.1982479 29.9581108  7.6496283
m62b  3.013072e+01  97.55556  7.731933 9  17.3153020 41.6760113 28.8832568
m64   1.963425e+01  76.11111  6.622327 9   5.3610793 27.5914637 17.5575755
m65   2.995183e+01  94.00000 10.381793 9  17.7987519 46.4316477 21.1652470
m67  -5.227316e+00  13.00000  4.121326 9 -12.6088217  1.1929902 -7.8749956
m72   6.841847e+00  48.66667  5.787346 9   0.8535060 15.0611051  2.7054818
t17a -2.911800e+00  18.55556  2.049158 9  -6.0677021  0.2905238 -4.4103875
t2    1.727719e+01  68.75000  9.185098 8   0.4792358 29.6883085 13.5795700
t24   2.916443e+01  93.55556  9.038167 9  17.2888703 45.9902224 21.1609913
t30  -2.371655e+00  17.88889  5.674301 9  -6.8711912 12.1890239 -4.8710630
t5    2.496937e+01  84.00000 10.206111 8  15.8956395 48.2306958 19.7437725
            Q50       Q75
C-   -0.3511888  1.663377
C+   37.0707307 49.411234
m61   5.8975442  8.581592
m62a 16.9989200 23.437463
m62b 30.4771354 32.044981
m64  21.8134376 22.397261
m65  28.8988830 31.204056
```

```
m67  -5.6101186 -2.169016
m72   3.4757178 12.745109
t17a -3.0529974 -1.733808
t2   16.6403971 22.849467
t24  29.2527347 33.397950
t30  -4.0739242 -2.720623
t5   23.0999781 25.332430

$comparison
NULL

$groups
     df_co$inhib groups
C+     112.83333      a
m62b    97.55556     ab
m65     94.00000    abc
t24     93.55556    abc
t5      84.00000     bc
m64     76.11111     bc
m62a    69.12500     cd
t2      68.75000     cd
m72     48.66667     de
m61     44.00000     de
C-      30.88889     ef
t17a    18.55556      f
t30     17.88889      f
m67     13.00000      f
```

b. <u>Against *F. oxysporum*</u>

Kruskal-Wallis rank sum test

Kruskal-Wallis chi-squared = 100.76, df = 13, p-value = 1.183e-15

LSD test

```
$statistics
     Chisq Df      p.chisq
  100.7584 13 1.221245e-15

$parameters
            test  p.ajusted    name.t ntr alpha
  Kruskal-Wallis bonferroni df_fo$ind  14  0.05

$means
```

```
       df_fo.inhib      rank       std r         Min       Max        Q25
C-    3.248270e-15  20.62500  5.130620 8  -7.0623302  8.070158 -2.6006379
C+    7.822971e+01 120.00000  4.886963 7  71.9376289 83.621019 74.0135279
m61  -3.446486e-01  20.44444  6.870899 9 -10.7458389 12.296878 -2.4119882
m62a  3.570038e+01  88.77778  6.944061 9  22.4727278 46.129394 33.1740872
m62b  3.873571e+01  94.22222  7.821053 9  25.5014057 48.706275 35.6497295
m64   3.238893e+01  81.22222  5.105710 9  23.2755864 39.971575 30.7910050
m65   3.836176e+01  93.88889  7.141493 9  25.3042748 45.358655 37.6046101
m67   1.377138e+01  48.22222 10.702874 9   0.4791977 29.667480  5.1625656
m72  -3.939295e+00  11.33333  3.615276 9  -7.8700417  3.166388 -6.5659803
t17a  7.265413e+00  36.66667  8.241150 9  -0.5621319 26.396514  1.1810113
t2    2.446182e+01  67.50000 10.031194 9   8.8948392 35.665227 14.1551867
t24   3.998572e+01  93.66667 13.364347 9  19.8422247 64.096775 28.4762677
t30   5.700583e+00  34.11111  6.538335 9  -1.1803160 20.188114  0.7237515
t5    2.367054e+01  65.61111 14.251264 9   8.8948392 54.567779 14.2847715
            Q50       Q75
C-   -0.8134965  2.696914
C+   80.3485138 81.836865
m61  -1.1984262  2.596927
m62a 35.6861878 38.699174
m62b 39.4930442 44.283927
m64  33.0089204 35.889866
m65  42.0932493 42.932024
m67  11.7645881 24.252736
m72  -5.7044747 -1.324134
t17a  5.6380545  9.901144
t2   28.0401623 31.024633
t24  42.1813537 47.033375
t30   4.5757837  6.666482
t5   18.9177091 29.043484

$comparison
NULL

$groups
     df_fo$inhib groups
C+     120.00000      a
m62b    94.22222     ab
m65     93.88889    abc
t24     93.66667    abc
m62a    88.77778    bcd
m64     81.22222    bcd
t2      67.50000    cde
t5      65.61111     de
```

```
m67     48.22222      ef
t17a    36.66667      fg
t30     34.11111      fg
C-      20.62500       g
m61     20.44444       g
m72     11.33333       g
```

c. Halo distance *C. orchidophilum*

Kruskal-Wallis rank sum test

Kruskal-Wallis chi-squared = 51.389, df = 6, p-value = 2.474e-09

LSD test

```
$statistics
   Chisq Df      p.chisq
  51.389  6 2.474246e-09

$parameters
            test p.ajusted    name.t ntr alpha
  Kruskal-Wallis      none df_co$ind   7  0.05

$means
     df_co.halo     rank       std r   Min   Max   Q25   Q50   Q75
m62a 0.07400000 16.44444 0.1530057 9 0.000 0.419 0.000 0.000 0.000
m62b 1.99533333 47.55556 0.6405431 9 0.791 3.038 1.739 2.036 2.340
m64  2.25422222 51.00000 0.5253508 9 1.535 2.918 1.866 2.370 2.780
m65  0.58822222 28.00000 0.4667675 9 0.000 1.232 0.326 0.407 1.025
t2   0.02866667 14.55556 0.0860000 9 0.000 0.258 0.000 0.000 0.000
t24  2.11233333 47.88889 0.7188978 9 1.113 3.552 1.695 1.841 2.390
t5   0.00000000 13.00000 0.0000000 8 0.000 0.000 0.000 0.000 0.000

$comparison
NULL

$groups
     df_co$halo groups
m64    51.00000      a
t24    47.88889      a
m62b   47.55556      a
m65    28.00000      b
m62a   16.44444      c
t2     14.55556      c
t5     13.00000      c
```

d. <u>Halo distance *F. oxysporum*</u>

Kruskal-Wallis rank sum test

Kruskal-Wallis chi-squared = 53.555, df = 6, p-value = 9.07e-10

LSD test

```
$statistics
     Chisq Df     p.chisq t.value      MSD
  53.55497  6 9.06994e-10 3.18283 10.34155

$parameters
            test  p.ajusted    name.t ntr alpha
  Kruskal-Wallis bonferroni df_fo$ind   7  0.05

$means
     df_fo.halo     rank       std r   Min   Max   Q25   Q50   Q75
m62a  0.1577778 16.22222 0.3130806 9 0.000 0.710 0.000 0.000 0.000
m62b  2.4307778 46.77778 0.8287884 9 1.111 3.256 1.947 2.744 3.186
m64   2.7718889 50.00000 0.3136393 9 2.354 3.258 2.603 2.685 2.947
m65   1.4163333 34.11111 0.6071594 9 0.530 2.321 0.882 1.276 1.870
t2    0.0000000 13.00000 0.0000000 9 0.000 0.000 0.000 0.000 0.000
t24   2.7513333 50.88889 0.7545678 9 1.452 3.676 2.129 3.136 3.190
t5    0.0000000 13.00000 0.0000000 9 0.000 0.000 0.000 0.000 0.000

$comparison
NULL

$groups
     df_fo$halo groups
t24    50.88889      a
m64    50.00000      a
m62b   46.77778      a
m65    34.11111      b
m62a   16.22222      c
t2     13.00000      c
t5     13.00000      c
```
